# Reducing Urban Greenhouse Gas Footprints

**DOI:** 10.1038/s41598-017-15303-x

**Published:** 2017-11-07

**Authors:** Peter-Paul Pichler, Timm Zwickel, Abel Chavez, Tino Kretschmer, Jessica Seddon, Helga Weisz

**Affiliations:** 1 Research Domain Transdisciplinary Concepts & Methods, Potsdam Institute for Climate Impact Research, PO Box 60 12 03, D-14412 Potsdam, Germany; 2 Center for Environment and Sustainability, Western State Colorado University, 600 N. Adams St., Gunnison, Colorado 81231 USA; 3 World Resources Institute, 10 G St NE #800, Washington, DC 20002 USA; 4 Department of Cultural History & Theory and Department of Social Sciences, Humboldt University Berlin, Unter den Linden 6, D-10117 Berlin, Germany

## Abstract

Cities are economically open systems that depend on goods and services imported from national and global markets to satisfy their material and energy requirements. Greenhouse Gas (GHG) footprints are thus a highly relevant metric for urban climate change mitigation since they not only include direct emissions from urban consumption activities, but also upstream emissions, i.e. emissions that occur along the global production chain of the goods and services purchased by local consumers. This complementary approach to territorially-focused emission accounting has added critical nuance to the debate on climate change mitigation by highlighting the responsibility of consumers in a globalized economy. Yet, city officials are largely either unaware of their upstream emissions or doubtful about their ability to count and control them. This study provides the first internationally comparable GHG footprints for four cities (Berlin, Delhi NCT, Mexico City, and New York metropolitan area) applying a consistent method that can be extended to other global cities using available data. We show that upstream emissions from urban household consumption are in the same order of magnitude as cities’ overall territorial emissions and that local policy leverage to reduce upstream emissions is larger than typically assumed.

## Introduction

Cities worldwide strive to reduce their greenhouse gas emissions. A plethora of city networks such as the Global Covenant of Mayors for Climate & Energy, C40, International Council for Local Environmental Initiatives (ICLEI), and the Global Parliament of Mayors have emerged to foster and motivate cooperation in cutting urban GHG emissions. In 2017, 7,500 cities worldwide, representing 685 million people, were signatories of the Global Covenant of Mayors for Climate & Energy (the largest of those networks) and have declared emission reduction targets^1^. The 20 major cities in the Carbon Neutral Cities Network have pledged to become climate neutral^2^. Non-state Actor Zone for Climate Action (NAZCA), the UNFCCC online platform which tracks the climate commitments of non-state actors, lists more than 2,500 cities and many more have pledged to reduce their emissions by joining a growing number of dedicated city networks^3^.

Although these initiatives increasingly recognize the inherent socio-metabolic openness of cities that inevitably leads to resource use and associated GHG emissions occurring outside the city boundaries^4,5^ most cities still focus their reduction efforts entirely on the emissions released directly from their territory^6^.

**Figure 1:**
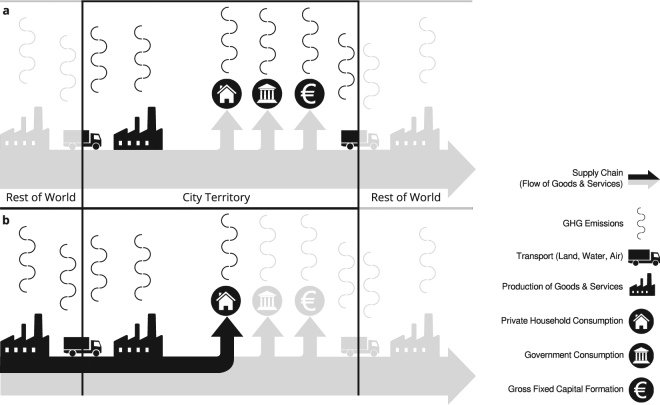
Conceptual comparison between territorial GHG emission accounting (**a**) and the GHG footprint (**b**). Territorial emissions include the entirety of emissions that occur within the city boundary. These are direct emissions from production (goods & services, transport) and final consumption (households, government, gross fixed capital formation). Because they also include urban production for exports, territorial emissions are often indicative of the economic structure of a city (e.g. in the presence of heavy industry). The GHG footprint, instead, puts the focus on consumption within the city boundary. In this study it includes direct and upstream GHG emissions from household consumption. The former occur within the city boundary (e.g. heating and private transport), the latter may occur anywhere in the world (including within the city) and require analysing the entire supply chain of urban consumption. The GHG footprint is indicative of the consumption pattern of urban households.

The vast majority of cities apply a perspective similar to the IPCC and OECD guidelines for national economies, where GHG emissions are attributed to the actors (households, firms, institutions) within the administrative territory on whose property or under whose legal control the emissions originate: e.g. the emissions from cement manufacturing are attributed to the cement producing company and the emissions from coal fired power plants to the electricity company. This traditional approach to allocate emissions is called territorial or production approach^4,7^. Figure 1a illustrates this for urban emissions. The territorial approach accounts for the direct emissions from all socio-economic actors within the city’s boundaries. There are the urban producers of goods and services and their associated transport (symbolized by a factory and a truck symbol in dark black in Fig. 1a). There are the final consumers, typically broken down into household consumption, government consumption and fixed gross capital (investments in durable goods, e.g. public infrastructure). It is important to note that economically we distinguish between two types of actors, producers and final consumers, whereas in GHG accounting all economic actors are producers of direct emissions (symbolized by undulate lines in Fig. 1).

A consumption perspective takes a different view^7^. As the name suggests, here emissions are attributed not to the economic producer but to the economic (final) consumer of a good or service. For example, emissions are attributed to the person consuming electricity at home rather than to the power company, or to the person eating a steak, rather than to the farmer who produced it. In summary, the emissions attributable to the goods and services purchased by local final consumers but occurring along the entire production chains of the purchased goods and services are called upstream or embodied emissions.

As global production chains continue to become longer and more complex, the difference between these two accounting perspectives has become considerable already at the national scale^8^. This observed separation of the geographic locations of production and consumption is even more pronounced for cities which are inherently open socio-metabolic systems. Today, urban upstream emissions often exceed those directly emitted on a city’s territory^9,10^. This fact limits the effectiveness of climate mitigation policies based on territorial emissions alone^11,12^.

The reasons why current urban climate change mitigation initiatives overwhelmingly focus on territorial emissions are both pragmatic and political. Local decision makers are often unaware of the relevance of upstream emissions. The literature on upstream urban emissions is sparse and comparisons among cities are hampered by differences in methods, classifications and terminology^4,13^. The large number of city networks, each with different guidelines for urban emission inventories contribute to this problem (see Supplementary Information online for more details on urban GHG accounting guidelines). With few exceptions, accounts of upstream urban emissions for cities in emerging countries are not available. Most importantly though, there is a widely held view that local politicians don’t have much policy leverage to influence emissions outside their own territory^14,15^.

To our knowledge this is the first international comparison of city specific GHG footprints from urban household consumption using a method that allows a near term, feasible and cross-city comparable inclusion of upstream emissions into urban GHG inventories. The GHG footprint is a composite indicator that combines direct emissions from local consumption sectors in the city with upstream emissions along global production chains attributable to local consumption. Different versions of GHG footprints are described in the literature^16^. In our study we calculate urban GHG footprints of household consumption, defined as the sum of direct and upstream GHG emissions associated with urban household consumption as defined for the national scale^8^ and illustrated for cities in Fig. 1b. In principle, it would be desirable to include the other local final consumption sectors, government consumption and gross fixed capital formation, into the urban GHG footprint. However, as city level data of these consumption sectors are not available, those sectors could only have been included using national averages scaled to the city level. Such an approach does not add any urban specific information and those two categories are therefore not considered in our GHG footprint accounts. However, to facilitate comparison with other studies we do provide national per capita averages for GHG emissions from government consumption and gross fixed capital formation alongside our city results.

We show that upstream emissions are relevant in cities in emerging and in developed countries and discuss ways in which local authorities could have substantial policy leverage to reduce both their territorial and their upstream emissions outside their geographic jurisdiction.

Upstream emissions of urban household consumption and GHG footprints have been calculated for a number of cities^14,16^. Most published studies report results for single cities^6,17,18^ or for multiple cities from a single country, e.g. UK^9^, Australia^10^, Finland^19^, USA^20,21^. With the exception of India^18^ and China^22,23^ studies for cities in developing or emerging economies are absent in the literature. The variation reported under the heading of urban GHG footprints is large ranging from 2.4 tCO_2_e/cap*yr (tons CO_2_ equivalents per capita and year) in Delhi to 60 tCO_2_e/cap*yr in Luxembourg^17,18^. It is important to note however, that different definitions of urban GHG footprints prevail in the literature (see Supplementary Information online for more details) therefore comparability between published results across different studies is very limited.

In addition to urban household footprints for individual cities, also aggregated national or regional urban household GHG footprints have been calculated^24–26^. This extremely important work serves a different purpose in providing statistically robust evidence of systemic patterns, such as persistent rural-urban differences or the dominant influence of income on GHG footprints of household consumption. This aggregated approach is, however, less useful for local policy that relies on site specific and comparable accounts, as the huge differences reported for individual cities clearly demonstrates.

We compare the upstream GHG emissions from household consumption of four global cities to their territorial emissions from all sources, show GHG footprints of their household consumption, investigate the geographic reach of their global hinterlands, and discuss leverage points for urban policy to reduce their territorial and upstream emissions. Berlin, Delhi NCT (National Capital Territory), Mexico City and the New York MSA (metropolitan statistical area) - four cities from three continents - were selected to represent different size, history, urban form, income level and national culture^27^.

To calculate upstream emissions we integrated data from household expenditure surveys for each of the four cities into Eora, a multi-regional input-output (MRIO) model with environmental extensions^28^. Our results are reported in tons of CO_2_ equivalents and include all Kyoto gases (CO_2_, CH_4_, N_2_O, HFCs, PFCs, SF_6_, NF_3_) for the year 2012 (2008 for Mexico City). Direct household emissions were taken from the respective local GHG emission inventories (see methods).

## Results

### Comparison between territorial and upstream emission

**Figure 2:**
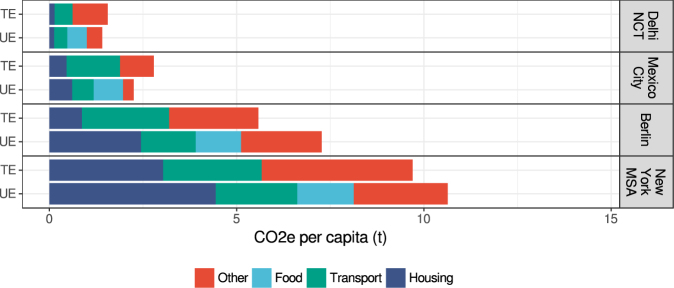
Sectoral comparison of total territorial emissions (TE) and upstream emissions of household consumption (UE) among the four cities in units of CO_2_e per capita per year.

The comparison between per capita total territorial emissions (TE) and upstream emissions from household consumption (UE) shows that they are of the same order of magnitude (Fig. 2, Table 1). New York MSA (9.7 tCO_2_e/cap*yr TE and 10.6 tCO_2_e/cap*yr UE) and Berlin (5.6 tCO_2_e/cap*yr TE and 7.3 tCO_2_e/cap*yr UE), the two more affluent cities, have much larger per capita emissions from both accounting perspectives compared to Mexico City (2.8 tCO_2_e/cap*yr TE and 2.3 tCO_2_e/cap*yr UE) and Delhi NCT (1.6 tCO_2_e/cap*yr TE and 1.4 tCO_2_e/cap*yr UE). Upstream emissions from household consumption are substantial, ranging between 81% (Mexico City) and 130% (Berlin) of territorial emissions; and in the two more affluent cities (Berlin and New York) they surpass territorial emissions.

**Table 1:** Upstream and direct household GHG emissions, GHG footprint and total territorial GHG emissions per sector [tCO_2_e/cap*yr].

City	GHG emission indicator (tCO_2_e/cap*yr)	Housing	Transport	Food	Other	Total
Berlin	Upstream (UE)	2.4	1.5	1.2	2.1	**7**.**3**
	Direct Household	0.9	0.8	—	—	**1**.**6**
	GHG Footprint	3.3	2.2	1.2	2.1	**8**.**9**
	Territoral (TE)	0.9	2.3	—	2.4	**5**.**6**
Delhi NCT	Upstream (UE)	0.1	0.4	0.5	0.4	**1**.**4**
	Direct Household	0.2	0.3	—	—	**0**.**5**
	GHG Footprint	0.3	0.7	0.5	0.4	**1**.**9**
	Territoral (TE)	0.2	0.5	—	0.9	**1**.**6**
Mexico City	Upstream (UE)	0.6	0.6	0.8	0.3	**2**.**3**
	Direct Household	0.2	0.6	—	—	**0**.**8**
	GHG Footprint	0.8	1.2	0.8	0.3	**3**.**1**
	Territoral (TE)	0.5	1.4	—	0.9	**2**.**8**
New York MSA	Upstream (UE)	4.4	2.2	1.5	2.5	**10**.**6**
	Direct Household	1.8	1.8	—	—	**3**.**6**
	GHG Footprint	6.2	4.0	1.5	2.5	**14**.**2**
	Territoral (TE)	3.0	2.6	—	4.0	**9**.**7**

### Sectoral composition of territorial emissions

The main sources of territorial emissions in the four cities are thermal services (space and water heating, cooking) in buildings and transport (Fig. 2). Building-related direct emissions are primarily determined by the living space per capita, thermal quality of the building stock, the heating technologies in use (e.g. on-site fuel combustion, district heating or electric heating) and the local climate^27^. Direct transport emissions are determined mainly by the emission intensity of the vehicle fleet and the share of private motorized trips in the modal split (i.e. the relative shares of different modes of transportation). The modal split, in turn, is influenced by urban form (including public transport infrastructure) and gasoline prices^29^. Territorial emissions summarized in the category “Other” include emissions from industry, commerce and public infrastructure services. Their share is highly variable and reflects the economic structure of a city (especially the presence of heavy industry)^4,9^.

### Sectoral composition of upstream emissions

The average shares across the four cities are 28% for housing, 23% for transport, 26% for food, and 24% for all other sectors. Thus, housing and transport contribute over 50% to the upstream emissions from household consumption. Together with food those three sectors account for three quarters of total upstream emissions, while all other consumer goods and services contribute only one quarter.

Upstream emissions in the housing category include those from electricity generation, remote heating, water supply, sewage and solid waste treatment, operational services to collect rent and provide accommodation and home maintenance and repair. Upstream emissions from transport include the production and maintenance of private cars as well as extraction, refining and transportation of gasoline, but exclude direct emissions from the operation of private vehicles. It also includes all emissions from private use of air travel, train, bus and other forms of public transport (including emissions for production, operation and maintenance of vehicles). Upstream emissions from food include production, processing and transportation of food items purchased by urban dwellers and emissions from visits to restaurants (see method section for a detailed breakdown of consumption categories).

### Urban household consumption GHG footprints

**Figure 3:**
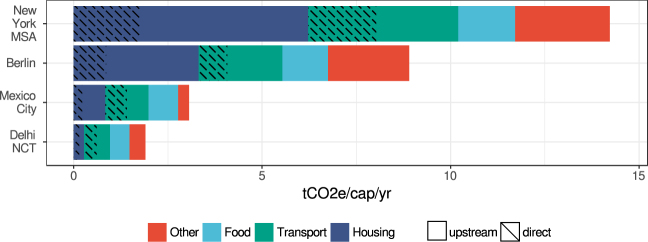
Per capita urban GHG footprints of the four cities and their sectoral composition. The shares of direct emissions in the footprint are indicated by criss-cross lines.

The estimated GHG footprints are Delhi NCT 1.9, Mexico City 3.1, Berlin 8.9 and New York MSA 14.2 tCO_2_e/cap*yr (Fig. 3). The share of direct household emissions in the footprint is 25% in Delhi, Mexico City, and New York MSA and 18% in Berlin. Housing, transport and food are responsible for over three quarters of the GHG footprints of all cities. The upstream emissions attributable to all other consumer goods (e.g. electronics, clothes, etc.) and health services purchased by urban dwellers only make up between 9–24% of the GHG footprint.

Using the GHG footprint to account for urban household emissions offers two main benefits over a simple comparison between total territorial and upstream emissions as shown in Fig. 2. Firstly, core infrastructure for electricity, heat, water and waste treatment is often situated at the urban periphery. Depending on whether such infrastructure is within or without the administrative territory of a city leads to notorious distortions in territorial emission accounting^30^. Secondly, territorial emissions and upstream emissions are not additive. Double counting would occur whenever parts of the supply chain of goods purchased in the city lie within the territorial boundary (e.g. the emissions of a district heating plant in a city are counted towards its territorial emissions as well as the upstream emissions of households). The GHG footprint used in this study resolves both of these issues.

### Geographical reach of upstream emissions

Besides knowing the sectoral composition of the GHG footprint, urban policy makers can also benefit from knowing its geographic reach. Tracing the geographic locations of upstream emissions gives evidence on how “global” the supply chains of contemporary cities have become.

**Figure 4:**
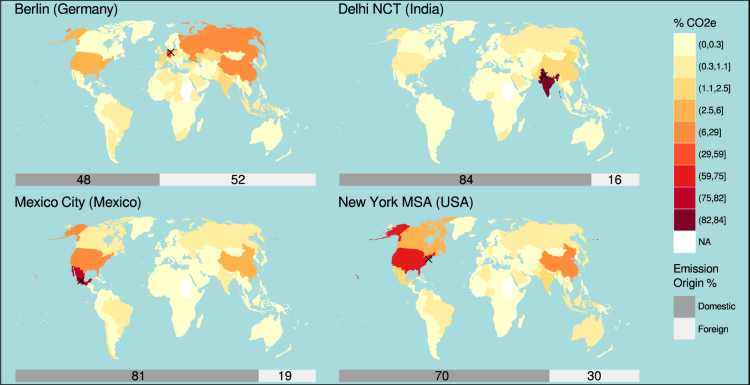
The global reach of urban GHG footprints. The four maps show the spatial distribution of the cities’ non-domestic upstream household GHG emissions. Maps are based on the Natural Earth public domain data set (http://naturalearthdata.com/) and were created in R^75^ using the ggplot2^78^ package.

Figure 4 shows that the shares of non-domestic upstream emissions range between 16% (Delhi NCT) and 52% (Berlin). Domestic refers to the nation state in which the city is located. The sample size of four certainly does not permit any generalizable conclusions on the global reach of urban supply chains but the results in Table 2 support the plausible hypothesis that the supply chains of wealthier cities (Berlin and New York MSA) are more international and those of cities in large nation states are more domestic. As shown in Table 2, the income effect seems to be particularly strong for food and “other” goods and services (primarily manufactures) where the domestic emission shares in Berlin (47% and 37%) and New York (53% and 44%) are considerably lower than in Delhi (79% and 88%) and Mexico City (75% and 51%). Note, that the high shares of domestic emissions in Delhi and Mexico City still correspond to much lower absolute domestic emissions compared to Berlin and New York (see Table 2). The comparatively low share of domestic upstream emissions in all sectors in Berlin is partly attributable to the European single market and partly to the large energy imports (mainly from Russia) in Germany’s domestic energy supply. The high share of domestic upstream emissions in the housing and transport sector of New York might be attributable to the large size of the US economy. More details about the regional and sectoral distribution can be found in the Supplementary Information online.

**Table 2:** Domestic shares in % of upstream emissions of urban households overall and in different consumption sectors (absolute values in tCO_2_e/cap*yr in brackets).

	Berlin	New York MSA	Delhi NCT	Mexico City
Housing	58.0 (1.4)	87.1 (3.9)	93.2 (0.1)	93.7 (0.6)
Transport	48.4 (0.7)	73.4 (1.6)	84.1 (0.3)	87.8 (0.5)
Food	46.6 (0.6)	53.1 (0.8)	78.7 (0.4)	74.6 (0.6)
Other	36.8 (0.8)	43.7 (1.1)	87.6 (0.4)	51.0 (0.1)
Overall	47.9 (3.5)	69.2 (7.4)	84.0 (1.2)	80.1 (1.8)

## Discussion

This study for the first time presents urban household GHG footprints for four cities from three continents using a consistent and widely applicable method for urban consumption based GHG accounting. This is an important step toward creating the basis for comparable benchmarking, which in turn can enable more effective city collaboration and competition to reduce urban GHG footprints.

Lack of methodological and terminological standardization, differences in data utilization, unequal inclusion of different GHGs, and differences in the definition of a GHG (or a carbon) footprint concept have so far prevented a meaningful comparison of urban GHG footprints between different studies (see also Supplementary Information online).

Previous studies conducted for the same cities calculated GHG footprints at 2.4 tCO_2e_/cap*yr for Delhi^18^ (Delhi NCT 1.9 tCO_2_e/cap*yr, this study), between 15 and 16 tCO_2_e/cap*yr for Berlin^31^ (8.9, this study), and ~16 tCO_2_e/cap*yr for New Yorks^32^ (14.2 New York MSA, this study).

Our results are consistently lower than those reported in the studies above. The main reasons for the discrepancies are fundamental differences in core definitions, methods, data sources, base years, and partly in geographic area of analysis (e.g. New York City vs New York MSA).

The Berlin results were obtained from national consumption data downscaled to the regional level, applied to standard households, using a different input-output model and bottom-up calculations of emission intensities, the details of which are not sufficiently described in the publication. In addition the authors report that their values are approximately 27% higher compared to estimates by the German Ministry of the Environment^31,33^.

The Delhi study^18^ applied a different footprint concept, originally called transboundary infrastructure supply chain footprint^34^, that extends territorial accounting by selected upstream emissions for key infrastructure services and materials (such as electricity, water supply, air transport, cement etc.) provided to the city from outside its territory. Thus, a meaningful comparison to the household consumption footprint calculated here is not possible. Conceptually infrastructure supply chain footprints should be compared to territorial accounts, as the latter is a subset of the former. Because we took the territorial GHG accounts for Delhi directly from^18^ this comparison simply repeats that Delhi’s territorial GHG emissions of 1.6 tCO_2_e/cap*yr make up 68% of the extended supply chain footprint of 2.3 tCO_2_e/cap*yr (see Supplementary Information online for more information about different urban GHG accounting approaches).

Apart from the peculiarities of the different methods and definitions applied in the literature our results are certainly low end estimations, due to the exclusion of governmental expenditures and gross fixed capital formation in our GHG footprint calculations. Although not specific for the urban scale, we computed per capita upstream GHG emissions for governmental expenditure and gross fixed capital formation for the national level, to provide a first estimate of their scale. The national averages for these two categories are 0.2 and 0.7 for India, 1.2 and 2.8 for Germany, 2.7 and 3.3 for the US, and 0.2 and 1.0 for Mexico, respectively (all in tCO_2_e/cap*yr).

Our method complies with the proposed British standard (PAS 2070)^35^ and conceptually follows a well-established definition of the GHG footprint on the national level^8^. Eora is a freely available environmentally extended multi-regional input-output model used internationally^28,36^. This allows cities to calculate and monitor their household GHG footprint provided direct emissions inventories and urban consumer expenditure surveys are available. At present, the necessity to reconcile incompatible data sources (consumer expenditure data and national input-output tables) requires considerable time effort and the guesswork involved introduces uncertainty into estimated urban GHG footprints. This could be overcome by improved reporting on the urban level and by efforts to harmonize local and national accounting systems. Additional uncertainty comes from the simplifying assumptions in input-output modeling, the given sectoral and spatial resolution of Eora, and uncertainty induced by the balancing algorithm applied in Eora. These are discussed in more detail in the method section.

Two further amendments to the presented method should be noted that would greatly improve the policy relevance of GHG footprints. Firstly, as discussed above, the present study considers only emissions from household consumption and disregards emissions for government consumption and investments (gross fixed capital formation). This gap could be closed by collecting city specific data for these two items and would allow the GHG footprint to capture the GHG emissions attributable to government and construction activities, the latter of which were shown to be substantial, particularly in the rapidly growing cities in emerging economies^37^. Secondly, the reliance on national input-output tables implies assuming uniform emission intensities in each sector across the national territory. Thus local efforts to supply low carbon goods and services are hidden in national averages. Ideally this problem could be addressed by providing local scale environmentally extended input-output tables^17,38^. However, it is unlikely that local input-output tables will be widely available and comprehensibly integrated into existing MRIOs any time soon. This creates a trade-off between widely adopting a method which can deliver comparable GHG footprint estimates for a large number of global cities and the necessary and continued efforts of the scientific community to increase the resolution and precision of state-of-the-art MRIO systems^38^. The urgency to substantially reduce GHG emissions in all parts of society, however, suggests that we begin with feasible extensions of urban GHG inventories immediately so that the perfect does not become the enemy of the good. We suggest that the method applied in this study is a reasonable balance between the conflicting goals of holistic urban GHG accounting and its near-term feasibility and the accuracy of urban specific GHG inventories and studies.

Most importantly, though, our results suggest that urban leaders can, in fact, influence some of the main sources of extraterritorial upstream emissions. It is challenging to directly regulate household consumption choices but several aspects of city policy affect them indirectly. Housing and transport are the main sources of direct household emissions of urban citizens. The same two consumption categories are also responsible (together with food) for the majority of upstream emissions. This commonality suggests that local policies can be effective at reducing both.

Transport policies aimed at direct urban emissions include land use and zoning policies to encourage higher-density settlements as well as a range of incentives to influence the modal split towards public transport, cycling or walking. Many of the specific measures, such as higher fuel prices or congestion taxes, fast and affordable public transport, or urban planning that encourages walking and cycling also incentivize fewer and smaller vehicles, thus reducing the upstream emissions of the car fleet. The upstream emissions of public transport infrastructure are directly amenable to local policy intervention. Urban public transport policies could, in addition to focussing on low operational energy and GHG emissions, include low carbon materials for public transport fleets and infrastructure into their climate mitigation goals. Cities’ options certainly depend on the supply of such alternatives, but with continuing volatile prices in global commodity markets^36^ and growing concerns about the criticality of mineral raw materials supply^39^, material-efficient vehicles with low carbon emissions in the production phase could become economically attractive goals also for the vehicle manufacturing industry.

The same logic applies to measures aiming to reduce emissions from housing. Building codes and construction standards that encourage energy efficiency in heating, cooling, and lighting also affect upstream emissions by influencing material choice. There are often trade-offs between the impacts of material choice on upstream and operational energy, but the point to note is that the policy lever for direct and upstream emissions associated with these aspects of housing is the same. Our findings suggest that it is important to revise policy goals based on lifecycle evaluation. “Zero waste” initiatives focused on reducing per capita environmental waste affect both direct and upstream emissions simultaneously. As in transport, city leaders have more direct control over the emissions from water and waste treatment services since these are often at least in part publicly financed. Finally, in parallel to market conditions motivating material efficiency and low carbon material choices, the rise and increasing ease of mobilisation of political protests against extraterritorial waste disposal may be a force against simply moving emissions outside the boundaries.

Deep emissions reductions in both sectors will require revising regulatory regimes and focusing them on metrics that acknowledge both direct and upstream emissions. Technology-forcing regulations based on progressively improving performance standards rather than technology prescription can be a significant driver of innovation, as demonstrated by the success of California’s climate mitigation policy^40^. As these are rolled out, however, the performance parameters need to be carefully and comprehensively designed.

Food consumption patterns seem less easily accessible for local policy. However, cities have some leverage via green procurement in public facilities (e.g. hospitals, schools, etc.). Considering that the livestock sector globally contributes 80% of all food related GHG emissions^41^, simply introducing or increasing vegan choices in communal catering could already have a significant impact. Further, a growing number of cities is starting to see the social, economic and ecological benefits of working more closely with their direct “hinterlands” (Sustainable Food Cities, Greenbelt Foundation), thereby reducing their food GHG footprint and strengthening local policy leverage.

To properly reflect the effects of such promising policies in GHG inventories of cities still requires considerable efforts in data collection and harmonization. We think that only by incorporating GHG footprints into their routine planning will cities have the ability and incentive to help overcome some of the practical limitations of current urban GHG accounting.

The Paris agreement of COP21 envisages cities as core elements in the UNFCCC process on climate change mitigation and many cities have already become committed and organized actors by joining city networks with a pledge to reduce GHG emissions^42^. The capacity and commitment of cities to act on climate change mitigation even in times of political uncertainty on the national and international level may prove essential to fulfilling the Paris agreement of keeping the increase of global mean temperature below the 2 °C guardrail. The method and results presented in this study provide an important first step towards internationally comparable benchmarking of the GHG footprints of cities and highlight why cities must both be encouraged and enabled to focus on their full emissions impact – upstream emissions as well as territorial emissions – as they continue to develop their climate mitigation plans.

## Methods

The GHG footprints of urban household consumption reported in this study include direct emissions from urban consumption activities (space and water heating, cooking, fuel use from combustion engines) and upstream emissions, i.e. global supply chain emissions attributable to the goods and services purchased by local consumers. They exclude emissions attributable to government services and capital investments for which city specific data was not available. Direct emissions from private consumption are based on local emission inventories and upstream emissions were calculated using local consumer expenditure surveys (CES) and environmentally extended multi-regional input-output modeling (Eora)^28^. Each section of the technical method description is preceded by a short non-technical summary.

### Upstream Emissions

CES data for Delhi NCT^43,44^, Berlin^45–47^, Mexico City^48,49^ and New York MSA^50^ were taken from local statistical sources. A number of pre-processing steps are necessary to make the raw CES data compatible to Eora. First, as the classification of consumption categories is different in each consumer expenditure survey and different between CES and the classifications used in Eora we constructed correspondence tables that map the corresponding categories of CES and Eora, simply called mapping in the remaining description. The classifications in Eora closely resemble the input-output tables provided by the national statistical offices of each country. Second, the local currency purchaser prices used in the CES are converted to USD base prices on a sector by sector basis. Finally, the import structure of urban final demand is mapped according to the national import structure.

#### Data Pre-processing

1. The raw CES data need to be transformed into a final demand vector that conforms to the respective sectoral structure used in the national section of each city’s home country in Eora. The correspondence between CES categories and Eora sectors is many-to-many, meaning that one CES category can correspond to multiple Eora sectors and vice versa. This correspondence was manually established with the help of various statistical sources that contain descriptions of the respective CES categories and Eora sectors (Eora sectors are similar but not always equivalent to the national accounts statistics reported by most countries). The statistical sources used were: Delhi NCT^51^/India^52^, Berlin/Germany^53,54^, Mexico City^55^/Mexico^56^, and New York MSA^57,58^/USA^59^. For each city-country pair (e.g. Berlin and Germany) the result of this process is a binary correspondence matrix *C*_*nxm*_, where *n* is the number of city CES categories and *m* is the number of national Eora sectors. An element *c*_*ij*_ is 1 if CES category *i* corresponds to Eora sector *j* and 0 otherwise. Given *C*, the system is still under-defined if we do not assume a uniform distribution between corresponding categories and sectors (e.g. expenditures in CES category “fruit” corresponds to Eora sectors “apples” and “pears” but not necessarily in equal shares). We assume the same ratio between corresponding categories/sectors (e.g. apples and pears) for urban and national final household demand. For this, we multiply each element in *C* column-wise with the Eora domestic final demand vector *Y* and normalize row-wise (dividing each element by its associated row sum) to arrive at a correspondence coefficient matrix C′. The city final demand vector *Y*′ can then be calculated as
  1 $${Y}^{^{\prime} }=x\ast {C}^{^{\prime} }$$
  where *x* is the CES vector of the city.
2. The CES data must be converted from local currency purchaser prices into USD base prices for use with the emission coefficients provided with in the Eora satellite accounts. Currency conversion was performed via World Bank official exchange rates^60^ which best reflect relative prices of tradeable goods^61^. Purchaser price (PP) to base price (BP) conversion was performed on a sector by sector basis using national BP/PP ratios calculated from Eora. Due to some inconsistencies in the data (i.e. negative purchaser prices or extremely high BP/PP ratios), base prices were capped at five times above or below purchaser prices (sensitivity analyses for factors 2 and 10 were performed).
3. In step 1 we have mapped all CES categories on domestic Eora sectors because CES data does not contain information on whether goods purchased in a city were produced in the domestic national economy or imported. We assume the urban import structure on a sector by sector basis to be equivalent to that on the national level. The practical problem presented by Eora is that it provides no correspondence tables between the heterogeneous national sector definitions across the 184 different countries (adding up to 14,838 sectors in total). While in principle the entire correspondence table could be constructed manually based on statistical sources, the large number of sectors in Eora makes this impractical. Instead, for each city’s home country we manually map those foreign sectors (sorted by decreasing size) that represent >90% of national final demand to 12 aggregate sectors (Agriculture, food, fossil fuel, manufacturing, furniture, electronics, paper, recreation, textiles, transport, health, housing). After also establishing correspondence tables between those 12 sectors and the domestic Eora sectors we obtain sectoral domestic/import ratios according to which we distribute city final demand *Y*′ across domestic and foreign sectors to generate the internationalized city final demand vector *Y*_*c*_ to be used in the input-output calculation.

#### Computing upstream emissions

Input-output tables are consistent, quantitative representations of the interlinkages (supply and use) among all production and final demand sectors within an economy, measured in monetary units. Eora, a multi-regional input-output model, represents the interlinked sectors of 184 countries (with a total of 14,838 sectors). Together with environmental extensions (i.e. of the total GHG emissions in physical units per sector), this allows to compute the upstream emissions along the entire supply chain for a given output (in USD) of any given sector.

The Leontief Total Requirements matrix (LTRM), also called the Leontief inverse, *L* is computed as

2 $$L={(I-A)}^{-1}$$

where *I* is the identity matrix and *A* is the technical coefficient matrix. A is calculated as

3 $$A=Z\ast {x}^{^{\prime} -1}$$

where *Z* is the inter-industry matrix and *x*′ is the total output vector *x* diagonalized into a matrix^62^.

Eora provides satellite data^63^ for each country and sector including annual emissions of Kyoto protocol greenhouse gases (GHGs) based on the Doha amendment^64^ (CO_2_, CH_4_, N_2_O, HFCs, PFCs, SF_6_, NF_3_). The satellite accounts for the F-gases provided by Eora had to be modified due to errors that are likely artefacts of Eora’s balancing and optimization algorithms. Instead of zeros, most cells uniformly contain 0.15 kt and some have negative emissions. Because overall F-gas emissions are very low but their conversion factors to CO_2_e are large, this leads to large distortions if uncorrected. The data was modified by subtracting 0.15 kt from each cell and replacing all negative values with zeros. This correction was successfully validated against national emissions reports^65^.

Non-CO_2_ gases are converted to CO_2_e using the common SAR GWP-100^66^ (and^67^ for NF_3_). The coefficient vector *k* (for CO_2_e emissions per USD and sector) is defined by dividing the annual emissions by total sectoral output. The coefficients *k*, the LTRM *L* and the city final demand vector *Y*_*c*_, yield the upstream emissions *e* in international sectoral resolution:

4 $$e=k\ast L\ast {Y}_{c}.$$

### Territorial emissions

Territorial emissions were taken from municipal GHG inventories. Not all cities reported all Kyoto gases at the same level of detail: Berlin^46^ (2012): CO_2,_ CH_4_, N_2_O; Mexico City^68^ (2012): CO_2_, CH_4_, N_2_O; NCT Delhi^18^ (2009): CO_2_, CH_4_, N_2_O). Direct emissions for New York MSA were gathered from four different greenhouse gas emission inventories (North Jersey^69^ (2006): CO_2_, CH_4_, N_2_O, HFCs, PFCs, SF, Long Island^70^ (2010): CO_2_, CH_4_, N_2_O, Mid-Hudson^71^ (Putnam, Rochester and Westchester counties only) (2010): CO_2_, CH_4_, N_2_O, HFCs, PFCs, SF_6_, New York City^72^ (2012): CO_2_, CH_4_, N_2_O, SF_6_).

The direct emission component in the GHG footprint includes only those territorial emissions emitted by the residents of the city. These include fuel use for space and water heating, cooking and private motorized transport. While those numbers could be taken directly from the GHG inventories of Delhi NCT and Mexico City, the statistical data for Berlin and New York MSA had to be disaggregated using additional sources from the literature.

For Berlin, overall road transportation emissions had to be disaggregated to private motorized transport emissions. The nationwide private vehicle emissions share of total road transport emissions in 2010 (74%) was taken from the Federal Environmental Agency (UBA)^73^ and multiplied by overall 2012 Berlin road transportation emissions.

Similarly, private motorized vehicle emissions in the North Jersey and Mid-Hudson counties were calculated by multiplying the light duty vehicle emissions share of overall road transportation emissions in the US in 2005 (75.37%) and 2010 (73.45%)^74^ with North Jersey and Mid-Hudson road transportation emissions, respectively. New York City’s taxi and for-hire car emissions (2006) share of total private motorized transport emissions (2005) (20.7%)^72^ was subtracted from total private motorized transport emissions (2012).

One overall caveat is that territorial transport emissions include local emissions by non-citizens (e.g. tourists) and exclude emissions of citizens outside the city.

### Result Aggregation and Visualization

For reasons of space and clarity, the detailed sectoral results for upstream emissions were aggregated into four categories for the presentation of the GHG footprint results. The composition of the four categories is:

- ***Food***: upstream: food, food away from home, non-alcoholic and alcoholic beverages, tobacco
- ***Housing***: upstream: rent/shelter, energy, maintenance of buildings, utilities; direct: heating and cooking using fossil fuels or charcoal/wood
- ***Transport***: upstream: purchase of cars, motor bikes, bikes, repair, maintenance, accessories, fuel, lubricants, public transportation, passenger transport, transport services, international transport, package holidays; direct: gasoline & diesel
- ***Other:*** upstream: services, health and all other manufactures; direct: industry, commerce, services, and government 

### Map Visualizations

All maps in Fig. 4. are based on the Natural Earth public domain data set (http://naturalearthdata.com/). The figures were generated in R^75^ using the rgeos^76^ and rgdal^77^ packages for geospatial calculations and ggplot2^78^ for visualization. The clusters of countries and associated ranges used in the world maps of Fig. 4 were defined according to Fisher-Jenks using the classInt^79^ package.

### Limitations and Uncertainties

The main goal of this study was to obtain methodologically consistent and comparable estimates of the GHG footprints of international cities using a state of the art method that can be extended to additional cities with reasonable effort. At present, the chief way to accomplish this is using MRIO models. However, these models (like all models) incorporate a number of simplifying assumptions and presently have restricted sectoral and geographical resolution. From this follow some caveats which lead to uncertainties in the GHG footprint estimates.

In input-output modeling, each sector is assumed to produce one uniform good (or a uniform basket of goods) at a uniform price and the same emission factor will apply to all goods within one sector of one country. For example, in the US input-output table, one dollar spent at an up-scale, locally sourced vegan restaurant in Manhattan will generate the same emissions as one dollar spent in an Alabama burger restaurant. Alleviating this problem requires not only input-output tables at higher sectoral and spatial resolution but also their integration into the MRIO model to accurately capture upstream emissions. Efforts to increase the sectoral and spatial resolution of MRIO models are currently underway^38^ but are unlikely to cover many parts of the world, particularly in developing countries, in the near future.

In addition to the model uncertainty in MRIO models, uncertainty is introduced into the GHG estimates through the mapping of CES to Eora as well as through the empirical uncertainty in the consumer expenditure data and all other statistical econometric and emissions data. The issue of mapping CES data to MRIO models can be addressed by local authorities by harmonizing their survey designs. The numerous sources of conceptual and empirical uncertainty call for a joint effort of the scientific community to develop a first quantification of the uncertainty range of urban GHG accounts.

### Data availability statement

The datasets generated during and/or analysed during the current study which are not publicly available and referenced above are available from the corresponding author on reasonable request.

## Electronic supplementary material

Supplementary information
