# Supplementary material for: Reducing Urban Greenhouse Gas Footprints
Source: Sci Rep. 2017 Nov 7;7:14659. doi: 10.1038/s41598-017-15303-x (PMC5676705; doi:10.1038/s41598-017-15303-x)
Supplement: Supplementary file 1 — Supplementary information [file 41598_2017_15303_MOESM1_ESM.pdf]

Reducing Urban Greenhouse Gas Footprints: Supplementary information

Peter-Paul Pichler[1)], Timm Zwickel[1)], Abel Chavez[2)], Tino Kretschmer[1)], Jessica Seddon[3)], Helga Weisz[1,4*)]

1) Research Domain Transdisciplinary Concepts & Methods, Potsdam Institute for Climate Impact Research, PO Box 60 12 03, D-14412 Potsdam, Germany

2) Center for Environment and Sustainability, Western State Colorado University, 600 N. Adams St., Gunnison, Colorado, USA 81231

3) World Resources Institute, 10 G St NE #800, Washington, DC 20002

4) Department of Cultural History & Theory and Department of Social Sciences, Humboldt University Berlin, Unter den Linden 6, D-10117 Berlin, Germany

*) Corresponding author: Helga Weisz (weisz@pik-potsdam.de)

1. Additional results

Core characteristics of the four cities

**City**	**Population (Millions)**	**Area (km^2)**	**Population Density (Pop/km^2)**	**Household Expenditure (USD/capita) Purchaser Prices**
Delhi NCT	17.1	1,483	11,523	729
Mexico City	8.8	1,485	5,961	4,078
Berlin	3.4	892	3,784	20,295
New York MSA	19.1	17,319	1,104	23,381

Table S1: Core characteristics of the four cities: Data are for 2012 except Mexico City, where data are for 2008. Sources: population[1–4], area[5–8], household expenditure own calculations based on[9–12] and exchange rates from the World Bank[13].

Global and sectoral distribution of urban upstream GHG emissions

The following 5 figures show the geographical and sectoral distribution of urban upstream emissions. The only difference between figure 4 and figure S1 is that in figure S1 the domestic emissions are not shown. As the domestic part is usually the largest part this allows for more contrast in the color coding of international results. The four remaining figures (S2-S5) showing the sectoral and geographical disaggregation of results follow the same principle.

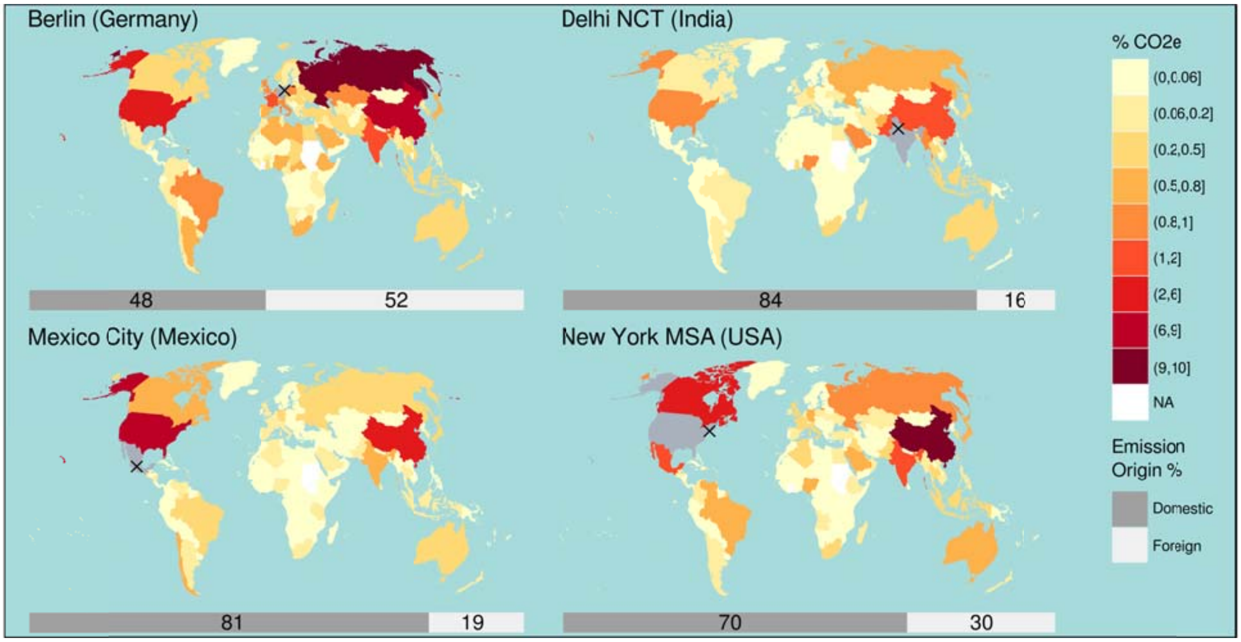

Figure S1: *The global reach of urban upstream emissions. The four maps show the spatial distribution of the cities' non-domestic upstream household GHG emissions. Maps are based on the Natural Earth public domain data set (http://naturalearthdata.com/) and were created in R*[14] *using the ggplot2*[15] *package.*

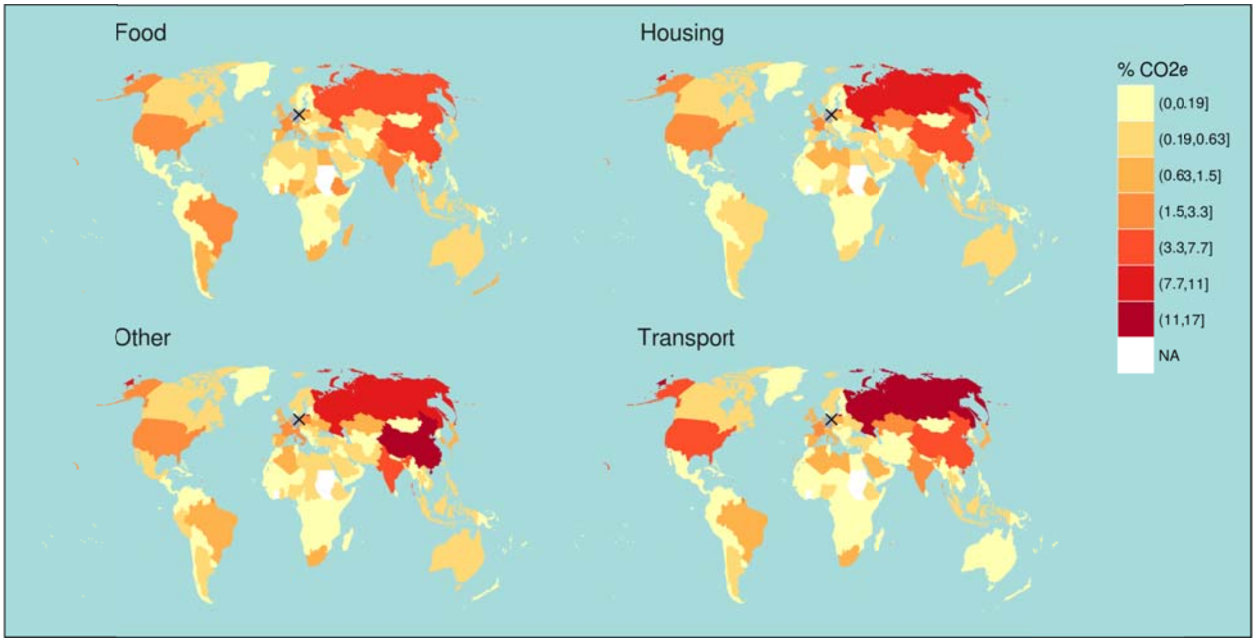

Figure S2: Berlin *sectoral and geographical disaggregation of non-domestic upstream household GHG emissions. Maps are based on the Natural Earth public domain data set (http://naturalearthdata.com/) and were created in R*[14] *using the ggplot2*[15] *package.*

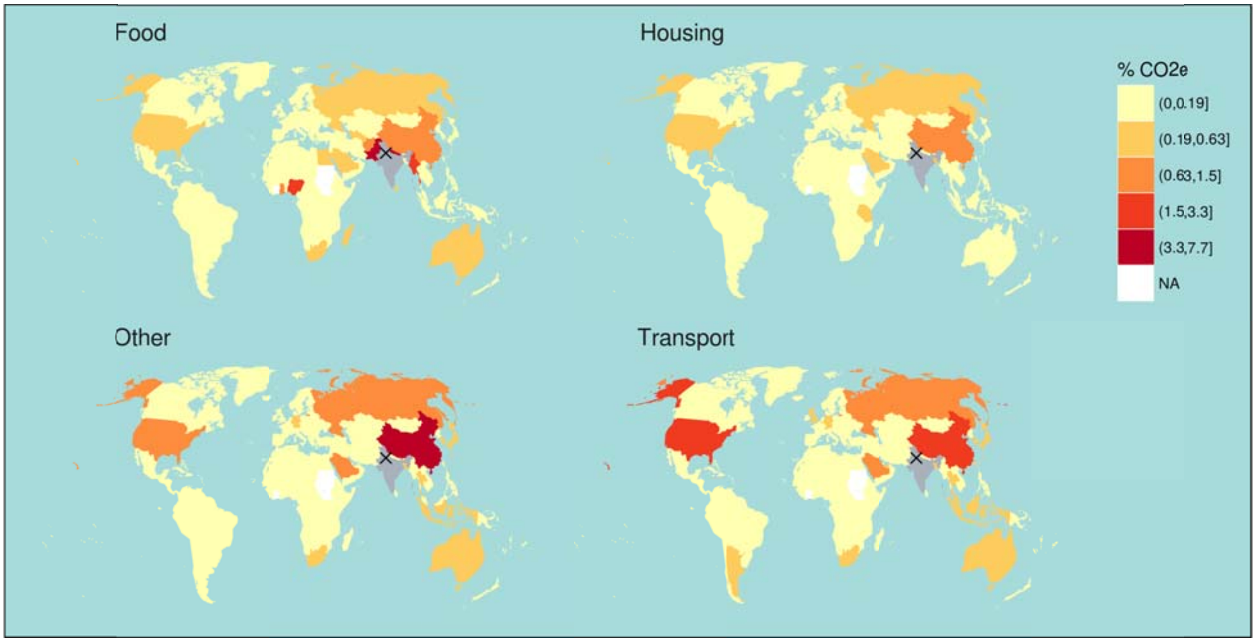

Figure S3: Delhi NCT *sectoral and geographical disaggregation of non-domestic upstream household GHG emissions. Maps are based on the Natural Earth public domain data set (http://naturalearthdata.com/) and were created in R*[14] *using the ggplot2*[15] *package.*

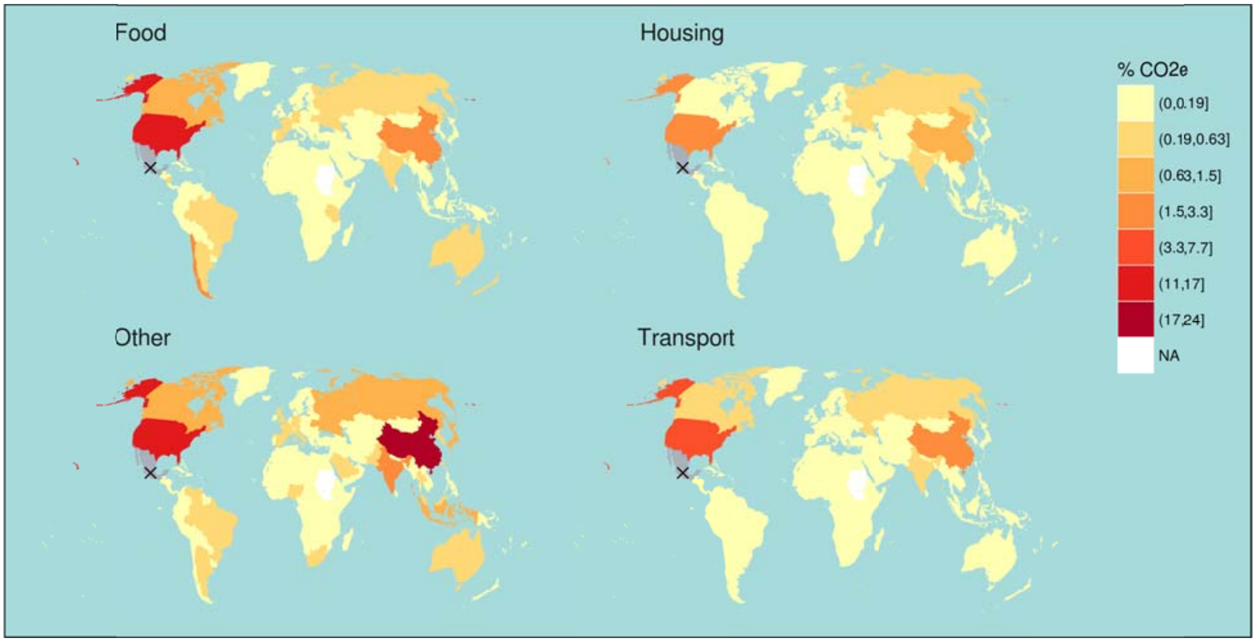

***Figure S4: Mexico City** sectoral and geographical disaggregation of non-domestic upstream household GHG emissions. Maps are based on the Natural Earth public domain data set (http://naturalearthdata.com/) and were created in R[14] using the ggplot2[15] package.*

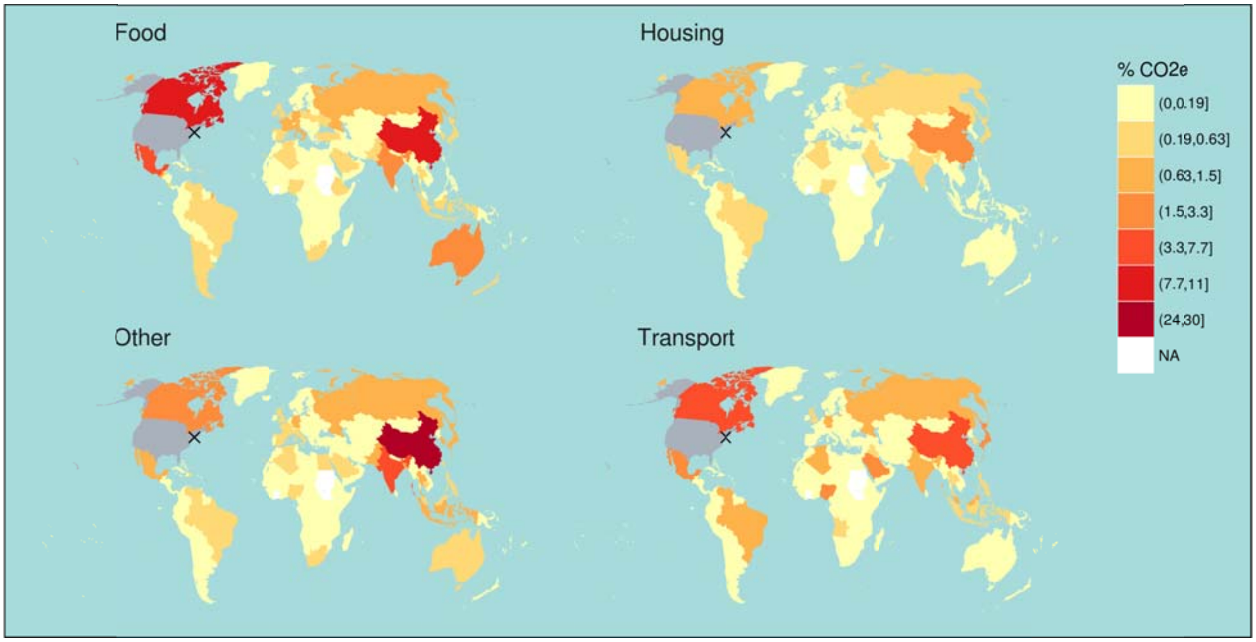

***Figure S5: New York MSA** sectoral and geographical disaggregation of non-domestic upstream household GHG emissions. Maps are based on the Natural Earth public domain data set (http://naturalearthdata.com/) and were created in R[14] using the ggplot2[15] package.*

2. Additional Background information and discussion

Urban GHG accounting approaches and terminologies

Three broad approaches to urban GHG emissions accounting can be distinguished[16]:

Territorial emissions accounting as applied in this study comprises of all emissions originating from socio-economic activities within the city's territory. Alternative terms for the same approach, that can be found in the literature are: direct emissions, scope 1 emissions, production approach, in-boundary emissions or purely geographical emissions. The degree of standardization and comparability of the territorial accounts is fairly good. The main sources of uncertainties are: different inclusions of green-house-gases, uncertainties in the statistical data, different definitions of urban system boundaries, and different classifications used for the sectoral disaggregation of urban activities.

Consumption based emissions include direct emissions for local final consumption plus all upstream emissions attributable to local consumption. Alternative terms used for upstream emissions are indirect, embodied, embedded, supply chain, production chain emissions. The sum of direct and upstream emissions attributable to the city's consumption sectors is called consumption based GHG (or carbon) footprint. Principle sources of uncertainty are the same as for territorial accounts, but additional uncertainties are introduced by the chosen allocation model (i.e. the specific input-output, LCA or hybrid input-output/LCA model) and its inherent uncertainties, and by varying inclusions of final consumption sectors. Uncertainties are thus higher than for territorial accounts, but can be limited by reporting explicitly which consumption sectors are included and by presenting sectoral results in an available standardized disaggregation (household consumption, government consumption, gross fixed capital). In our paper we suggest that a combination between the multi-regional input-output model EROA, which has the highest available sectoral and regional resolution, and a focus on urban household consumption currently represents the best option for near term, widely applicable and comparable urban GHG footprints.

Territorial plus upstream emissions of key infrastructures: This approach was first proposed in[17] and applied to Denver, Colorado. It starts from territorial urban emission accounts and adds upstream emissions associated with various key infrastructures and materials that serve the city but occur outside the city's territory, such as e.g. airports, commuter travel, and upstream emissions of materials imported to the city (such as water, fuels, cement etc.). In this approach the added upstream emissions are often called extraterritorial or trans-boundary emissions. The resulting composite indicator was originally termed "transboundary infrastructure supply chain footprint", but other names can be found in the literature[18,19] Following the published literature we can see that the allocation models used for estimating the upstream, extraterritorial emissions vary from city to city, as well as the included materials and infrastructures. Later extended indicators, such as e.g. "production based footprint" and "purely production footprint" have been proposed[19]. Clearly many more indicators could in principle be constructed, and some might be useful for local politicians. The main sources of uncertainty of the territorial plus infrastructures approach are the same as for territorial and consumption based accounting, but the lack of a comprehensive and consistent system boundary definition (i.e. a comprehensive list of infrastructures and materials to be included) adds additional uncertainty. Despite the fact that the consumption approach and the territorial plus infrastructure approach lead to composite indicators which are both denoted as "footprint", the conceptual differences between the two are so fundamental that a meaningful comparison of the numerical results is not possible.

Urban GHG reporting and initiatives

1. Urban GHG reporting

Urban GHG reporting protocols fall into three camps, using various versions of the principle accounting approaches described above and different terminologies.

1. Those for territorial emissions (e.g. the Baseline Emissions Inventory/Monitoring Emissions Inventory developed for the Covenant of Mayors).
2. Those for territorial and extraterritorial emissions associated with infrastructure services used in the city. The International Local Government GHG Emissions Analysis Protocol

(IEAP)[20] developed by ICLEI in 2009, for example, focuses on territorial emissions plus extraterritorial emissions associated with government operations. Its successor, the Global Protocol for Community-Scale Greenhouse Gas Emission Inventories (GPC)[21] developed by C40, the World Resources Institute, and ICLEI, divides emissions into three "scopes". Scope 1 emissions are territorial, Scope 2 are related to grid-based energy used in the cities, including emissions released outside the city boundary, and Scope 3 includes extraterritorial emissions stemming from activities taking place inside the city. Indirect emissions from household consumption are acknowledged, but not included in detail in the protocol. "The GPC includes Scope 3 accounting for a limited number of emission sources, including transmission and distribution losses associated with grid-supplied energy, and waste disposal and treatment outside the city boundary and transboundary transportation. Cities may optionally report other Scope 3 sources associated with activity in a city—such as GHG emissions embodied in fuels, water, food and construction materials. To support cities in measuring these and other scope 3 emissions in a robust and consistent manner, the GPC authors *anticipate* providing additional guidance on estimating emissions from key goods and services produced outside the city boundary"[21] (p. 33, emphases added).

3. Those for territorial emissions, infrastructure-related emissions inside and outside boundaries, and consumption-related emissions. These include IPCC International Standard for Determining Greenhouse Gas Emissions for Cities (ISDGC), the U.S. Community Protocol for Accounting and Reporting of Greenhouse Gas Emissions, and PAS 2070: Specification for the assessment of greenhouse gas emissions of a city.

The major city collaboration platforms tend to focus mainly on territorial emissions, using subsets of these protocols. Scope 3 emissions in particular create huge problems for comparability, as the protocols leave it open which infrastructures are included. Territorial emission and consumption based household footprints provide better comparability, but also for those accounting approaches variations exists, that should be made explicit.

The Covenant of Mayors, one of the largest platforms for cities to disclose their action plans and report on progress in GHG mitigation, uses a specially developed baseline emissions

inventory focused on territorial initiatives. In the GPC, used among the C40 cities to report on progress in GHG emissions, cities have the choice of two reporting options: BASIC and BASIC+. BASIC covers territorial emissions from stationery energy and transportation (Scope 1), extraterritorial emissions related to grid-supplied energy (Scope 2), and emissions related to waste inside and out of the city boundaries (Scope 3). Other extraterritorial emissions stemming from activities inside the city, such as extraterritorial transport, transmission and distribution, other indirect emissions are not included. BASIC+ includes territorial emissions from industrial processes and agriculture/land use within the city boundaries, as well as transboundary transportation. Of the 56 C40 cities that use GPC to report GHG emissions, only two (Auckland and Mexico City) use the BASIC+ guidelines. The others report "emission sources that occur in almost all cities (stationary energy, in-boundary transportation, and in-boundary generated waste)." [as stated on C40 website, accessed August 13, 2017: http://www.c40.org/other/gpc-dashboard]. Concluding, we can state that while these initiatives recognize the importance of upstream emissions and of policy collaboration across jurisdictions, they rarely count, report, or expose a complete picture of those emissions, when evaluating cities' progress.

To the extent that these initiatives' theory of change is correct - that counting, transparency about plans and achievements do create pressure and competition for city leaders to mitigate GHG - they are essentially seeking an incomplete, potentially misleading change. Ignoring upstream emissions from household consumption also runs the risk of enabling misleading correlations. As a simple thought experiment, suppose that wealthier cities are first to undertake infrastructure changes that significantly lower Scope 1 & 2 emissions. The correlation between wealth and the headline "GHG emissions" would be negative even as the GHG footprints of these cities could still be increasing.

References

1. Government of NCT of Delhi. *Level and Pattern of Household Consumer Expenditure in Delhi*. (Directorate of Economics and Statistics, 2012).

2. Consejo Nacional de Población (CONAPO). Población 2006-2012. (2015). Available at: http://www.conapo.gob.mx/es/CONAPO/Poblacion_2006-2012. (Accessed: 7th December 2016)
3. Amt für Statistik Berlin-Brandenburg. Statistisches Informationssystem Berlin-Brandenburg StatIS-BBB. *Statistisches Informationssystem Berlin-Brandenburg StatIS-BBB* (2016). Available at: https://www.statistik-berlin-brandenburg.de/statis/login.do?guest=guest. (Accessed: 7th December 2016)
4. US Conference of Mayors. *US Metro Economies (July 2012)*. (2012).
5. Government of NCT of Delhi. *Statistical Abstract of Delhi 2012*. (Directorate of Economics and Statistics, 2012).
6. Government of Mexico City. CDMX: Sobre nuestra ciudad (Mexico City: About our city). (2016). Available at: http://www.cdmx.gob.mx/cdmx/sobre-nuestra-ciudad. (Accessed: 7th December 2016)
7. Amt für Statistik Berlin-Brandenburg. *Die kleine Berlin Statistik 2015 - English edition*. (2015).
8. US Census Bureau. American FactFinder, USA Counties Data File Downloads. (2015). Available at: http://factfinder.census.gov/faces/nav/jsf/pages/searchresults.xhtml, https://www.census.gov/support/USACdataDownloads.html. (Accessed: 7th December 2016)
9. National Sample Survey Office, Ministry of Statistics and Programme Implementation, Government of India. *Household Consumption of Various Goods and Services in India 2011-12 (2011 data used: MPCEURP (Monthly per Capita Expenditure, Uniform Reference Period))*. (Government of India, 2014).
10. Instituto Nacional de Estadística y Geografía. *Encuesta Nacional de Ingresos y Gastos de los Hogares 2008*. (2009).
11. Amt für Statistik Berlin-Brandenburg. *Einkommen und Einnahmen sowie Ausgaben privater Haushalte im Land Berlin 2013*. (2015).
12. US Bureau of Labor Statistics. Consumer Expenditure Survey - Metropolitan Statistical Area (MSA) Tables 2012/2013. (2015).
13. World Bank. World Development Indicators (WDI) Database. (2016).
14. R Core Team. R: A language and environment for statistical computing. R Foundation for Statistical Computing (version 3.4.1), Vienna, Austria. (2017). Available at: https://www.R-project.org/.
15. Wickham, H. ggplot2: Elegant Graphics for Data Analysis (R package version 2.2.1). (Springer, 2009.
16. Seto, K. C.-Y. *et al.* Human Settlements, Infrastructure and Spatial Planning. in *Climate Change 2014: Mitigation of Climate Change. Contribution of Working Group III to the Fifth Assessment Report of*

the Intergovernmental Panel on Climate Change. Cambridge University Press, Cambridge, United Kingdom and New York, NY, USA. (2014).

17. Ramaswami, A., Hillman, T., Janson, B., Reiner, M. & Thomas, G. A Demand-Centered, Hybrid Life-Cycle Methodology for City-Scale Greenhouse Gas Inventories. *Environ. Sci. Technol.* **42,** 6455–6461 (2008).
18. Chavez, A. & Ramaswami, A. Progress toward low carbon cities: approaches for transboundary GHG emissions' footprinting. *Carbon Manag.* **2,** 471–482 (2011).
19. Lin, J., Hu, Y., Cui, S., Kang, J. & Ramaswami, A. Tracking urban carbon footprints from production and consumption perspectives. *Environ. Res. Lett.* **10,** 054001 (2015).
20. ICLEI. *International Local Government GHG Emissions Analysis Protocol*. (2009).
21. Fong, W. K. *et al. Global Protocol for Community-Scale Greenhouse Gas Emissions*. (WRI, C40, ICLEI, 2014).
